# Supplementary material for: Enhancement of Nitric Oxide Bioavailability by Modulation of Cutaneous Nitric Oxide Stores
Source: Biomedicines. 2022 Aug 29;10(9):2124. doi: 10.3390/biomedicines10092124 (PMC9496039; doi:10.3390/biomedicines10092124)
Supplement: Supplementary file 1 [file biomedicines-10-02124-s001.zip › biomedicines-1848426-supplementary.pdf]

biomedicines

Supplementary Material

Materials and Methods

UVA-induced Decomposition of Nitrite (Figure 1)

Aqueous solutions (in PBS, pH 7.4) of sodium nitrite at an indicated concentration were UVA irradiated by (70 mW/cm2) with or without sodium ascorbate (ASC). UVA irradiations of the solutions (in total 20 ml) were performed in a quartz glass cylinder (3.3 cm diameter, 120 cm^3), permanently flushed by carrier gas (nitrogen) for quantification of NO by chemiluminescence detection (CLD) exactly as described by us previously [1].

NO emanating from skin and analysis (Figure 1)

The methods to measure the effects of UVA on and NO release from skin were described previously [2]. A UVA Sellas-4000 lamp (Sellas Medizinische Geräte, Gevelsberg, Deutschland, 340-410 nm,; 18 mW/cm^2) was used as a UVA source for the experiments.

Characterization of NO-releasing Nitrite Solutions (Figure 2)

For characterization of NO release acetic/acetate buffer (pH 5.5, 500 µl,500 mM) containing sodium ascorbate (200 mM) and 500 µl of sodium nitrite solution (0.02%, 0,2%, 2.0%--> 2.9, 29, 290 mM) freshly prepared in milipore water were mixed and purged by carrier gas (nitrogen, 500 ml/min). Concentrations of released NO were measured in the gas flow by chemiluminscence detection (NO-analyzer CLD 822 Sr; Eco Physics, Duernten, Switzerland) and NO amounts/rates were calculated by integration of the resulting graphs (0–600 s).

Transdermal Penetration of Nitric Oxide released from Nitrite-containing Solutions (Figure 2)

The experimental set up is depicted in **A**. Human skin specimens were prepared as split-thickness skin grafts (thickness 0.3 mm), and mounted on custom made Franz diffusion cells, each between a donor chamber (37°C) and a receptor chamber. The surface area of each mounted skin graft was 0.785 $cm^{2.}$

1 ml nitric oxide releasing nitrite solutions (for preparation see above) were pipetted into the donor chamber. The receptor chamber was constantly purged by a nitrogen gas (100 ml/min) and the NO concentration was measured by chemiluminescence detection (CLD 88e; Eco Physics, Duernten, Switzerland). The penetrated amounts of NO were calculated by integrations of the resultant graphs (0–600 s).

Detection of Apoptosis *in sit*u (Figure 2)

Human skin specimens were obtained with patients' consent from mammoplastic or abdominoplastic surgery. The use of human material was approved by the local ethics committees of the Medical Faculty of the RWTH University Aachen (Votum No.EK163/07and the Medical Faculty of the Heinrich-Heine-University Düsseldorf (Study No. 3634). All experiments were conducted in compliance with the Declaration of Helsinki Principles. Micro-centrifuge tubes (Eppendorf, Hamburg, Germany), whose bottoms were cut off, were fixed on full-thickness skin using double-sided adhesive tape (LEA, Giessen, Germany) with defined holes (0.785 cm^2). Sodium nitrite and sodium ascorbate were prepared freshly as described above and directly mixed in the fixed tubes. After a 10 min incubation, solutions were aspirated and the treated areas were washed twice with PBS. Tubes were removed and by using a 8 mm biopsy punch (kai Europe, Solingen, Germany) samples were taken and cultured 24 h in 12 well cell culture dishes (Greiner, Frickenhausen, Germany) containing Dulbecco's modified Eagle'sMedium (DMEM) (Gibco-Invitrogen, Karlsruhe, Germany) supplemented with 10% fetal calf serum (FCS), 100 U/ml penicillin (Pen) and 100 µg/ml streptomycin (Strep) (PAA, Pasching, Austria) under standard culture conditions (37 C/5% CO2). Skin samples were fixed in 4% methanol-free formaldehyde solution in PBS, and prepared for TUNEL-assay [3]. Skin tissue sections (5 µm) and DeadEnd™ Fluorometric TUNEL System (Promega, Madison, WI, USA) were used according to the manufacturer´s protocol. DAPI-stained nuclei and apoptotic events were immediately analyzed under a fluorescence microscope (Axiovision, Zeiss, Germany).

Treatment with Dielectric Barrier Discharge (DBD) device (Figure 3)

Treatment with the DBD has been described elsewhere [4]. Briefly, non-thermal DBD plasma was produced and applied using a prototype device provided by Cinogy (Figure 1). Plasma was generated by applying an alternating polarity pulsed 120 hz voltage of ~14 kV magnitude (peak-to-peak). The DBD device was oper-ated in air for the treatment of human skin. For all DBD plasma applications, the distance between the DBD electrode and the biological sample was kept constant at 1 mm using a modified drill stand.

Detection of Nitrite, Nitrate and RXNO (Figures 1–3)

The concentrations of nitrite and nitrosated compounds (RXNO) were quantified by reductive denitrosation using a mixture of iodine/iodide in glacial acetic acid and the released NO was detected by the chemiluminescence reaction with ozone using the NO-analysator CLD 88 (Ecophysics, Munich, Germany) as described previously [5].

Quantification of Nitrit/Nitrate/RXNO in Skin Tissue (Figure 3)

The procedure for quantification of nitrite/nitrate/RXNO after DBD plasma application in humans skin specimens have been described elsewhere [6]

Measurements of Microcirculation (Figure 3)

Hairless skin of the forearm of 4 volunteers were treated with plasma for 90 s and microcirculation parameters such as blood flow and velocity, tissue perfusion, oxygen saturation, and blood filling of microvessels were assessed noninvasively using a microlightguide spectrophotometer (O2C; LEA Medizintechnik, Giessen, Germany) at time points indicated [7].

References

1. Suschek, C. V., A. Paunel, and V. Kolb-Bachofen. "Nonenzymatic Nitric Oxide Formation During Uva Irradiation of Human Skin: Experimental Setups and Ways to Measure." *Methods Enzymol* 396 (2005): 568-78.
2. Opländer, Christian, Annika Deck, Christine M. Volkmar, Michael Kirsch, Jörg Liebmann, Matthias Born, Frank van Abeelen, Ernst E. van Faassen, Klaus-Dietrich Kröncke, Joachim Windolf, and Christoph V. Suschek. "Mechanism and Biological Relevance of Blue-Light (420–453nm)-Induced Nonenzymatic Nitric Oxide Generation from Photolabile Nitric Oxide Derivates in Human Skin in Vitro and in Vivo." *Free Radical Biology and Medicine* 65 (2013): 1363-77.
3. Ben-Sasson, S. A., Y. Sherman, and Y. Gavrieli. "Identification of Dying Cells--in Situ Staining." *Methods Cell Biol* 46 (1995): 29-39.
4. Heuer, Kiara, Martin A. Hoffmanns, Erhan Demir, Sabrina Baldus, Christine M. Volkmar, Mirco Röhle, Paul C. Fuchs, Peter Awakowicz, Christoph V. Suschek, and Christian Opländer. "The Topical Use of Non-Thermal Dielectric Barrier Discharge (Dbd): Nitric Oxide Related Effects on Human Skin." *Nitric Oxide* 44 (2015): 52-60.
5. Feelisch, M., T. Rassaf, S. Mnaimneh, N. Singh, N. S. Bryan, D. Jourd'Heuil, and M. Kelm. "Concomitant S-, N-, and Heme-Nitros(Yl)Ation in Biological Tissues and Fluids: Implications for the Fate of No in Vivo." *FASEB J* 16, no. 13 (2002): 1775-85.
6. Paunel, Adnana N., André Dejam, Sven Thelen, Michael Kirsch, Markus Horstjann, Putrika Gharini, Manfred Mürtz, Malte Kelm, Herbert de Groot, Victoria Kolb-Bachofen, and Christoph V. Suschek. "Enzyme-Independent Nitric Oxide Formation During Uva Challenge of Human Skin: Characterization, Molecular Sources, and Mechanisms." *Free Radical Biology and Medicine* 38, no. 5 (2005): 606-15.
7. Beckert, S., M. B. Witte, A. Konigsrainer, and S. Coerper. "The Impact of the Micro-Lightguide O2c for the Quantification of Tissue Ischemia in Diabetic Foot Ulcers." *Diabetes Care* 27, no. 12 (2004): 2863-7.
